# Comparison of diffusion, cytotoxicity and tissue inflammatory reactions of four commercial bleaching products against human dental pulp stem cells

**DOI:** 10.1038/s41598-019-44223-1

**Published:** 2019-05-23

**Authors:** C. Llena, M. Collado-González, D. García-Bernal, R. E. Oñate-Sánchez, C. M. Martínez, J. M. Moraleda, F. J. Rodríguez-Lozano, L. Forner

**Affiliations:** 1 Department of Stomatology, Universitat de València, Valencia, Spain; 2 Unit of Special Care in Dentistry and Cell Therapy and Hematopoietic Transplant Unit, Internal Medicine Department, IMIB-Arrixaca, University of Murcia, Murcia, Spain; 3 Experimental Pathology Unit, IMIB-Arrixaca, University of Murcia, Murcia, Spain

**Keywords:** Dental biomaterials, Tooth whitening

## Abstract

Multiple side effects related to bleaching were found to occur in the dental pulp tissue, including decreased cell metabolism and viability. In this work we evaluated the *in vitro* diffusion capacity, cytotoxicity and biocompatibility of four commercial bleaching products on stem cells from human dental pulp (*h*DPSCs). Two commercial bleaching gels hydrogen peroxide-based (HP), Norblanc Office 37.5% (Nor-HP) and Opalescence Boost 40% (Opal-HP) were applied for 30 min to enamel/dentine discs. Another two gels from the same manufacturers, 16% carbamide peroxide-based (CP), Norblanc Home (Nor-CP) and Opalescence CP 16% (Opal-CP), were applied for 90 min. The diffusion of HP was analysed by fluorometry. Cytotoxicity was determined using the MTT assays, the determination of apoptosis, immunofluorescence assays and intracellular reactive oxygen species (ROS) level. Tissue inflammatory reactions were evaluated histopathologically in rats. Statistical differences were performed by one-way ANOVA and Bonferroni post-test (α < 0.05). Normon products showed lower cytotoxicity and diffusion capacity than the Ultradent products. A high intracellular ROS level was measured in *h*DPSCs after exposure to Opal-HP. Finally, a severe necrosis of both coronal and radicular pulp was observed with Opal-HP. Similar concentrations of hydrogen peroxide and carbamide peroxide in a variety of bleaching products exhibited different responses in cells and dental pulp tissue, suggesting that bleaching products contain unknown agents that could influence their toxicity.

## Introduction

In 1989, Haywood and Harald Heymann introduced vital tooth whitening with a research paper called ‘Nightguard vital bleaching’. The technique involved applying a gel (carbamide peroxide) as the subject slept, by means of a custom-made tray^1,2^. The paper opened up a new research line as many researchers looked into new techniques^3^, leading to the introduction of several new whitening products, although most used hydrogen peroxide (HP) or carbamide peroxide (CP) as their basis, applying the same directly by means of mouth guards or strips or simply directly applied^4^.

Furthermore, three bleaching approaches have been used: in-office bleaching, at-home bleaching, and a combination of both techniques. For at-home bleaching, studies recommend the use of low concentrations of bleaching agent (10–16% CP or 6% HP) applied for at least two weeks. For in-office procedures, bleaching agents (HP 25–40% or CP 35%) are applied for shorter time periods^5,6^.

In the case of HP, at least, the bleaching agent diffuses through the enamel and dentin to produce reactive oxygen species (ROS) that react with other free or weakly bound substances, whose molecular stability is subsequently re-established. This oxidant phenomenon may be the responsible of the mechanistic complexity of dental bleaching^7^. However, multiple side effects related to bleaching have been found to occur in the dental pulp tissue, including decreased cell metabolism and viability^8,9^, changes in vascular permeability^10^, DNA modifications^11^ and pulp necrosis^12–15^.

Dental pulp is a living tissue localized in the pulp chamber of the tooth, originating from the embryonic dental papilla (*i*.*e*., ectomesenchymal tissue)^16^. It has been reported that dental pulp plays a primary role in the regenerative response after an injury or trauma by secreting tertiary dentin and contributing to the differentiation of stem cells from dental pulp (DPSCs) into odontoblast-like cells^17,18^.

DPSCs are a progenitor population of cells with high proliferation and can differentiate into various cell lineages, e.g. osteoblasts-like cells, odontoblast-like cells, skeletal muscle cells, chondrocytes and adipocytes^19^. As DPSCs can be obtained from tooth under local anesthesia and without damage, they are the ideal seeding cells for medicine regenerative research and cytotoxic studies^20^.

*In vitro* cell cultures are an appropriate and frequently reported model for studying the deleterious effects of a variety of dental agents and/or materials on dental tissues^21^, as well as for studying the biological reactions of dental pulp cells to such materials. Although the results of *in vitro* studies cannot replace studies in humans, they are still regarded as a suitable way of testing new products and of ascertaining possible secondary effects^22^. In this respect, there several different methods and combinations of the same heve been studied in the case of whitening agents^23,24^.

Bleaching gels used in clinical treatment may contain HP concentrations of up to 40% causing different degrees of cell damage, although such damage was not observed with 16% CP concentrations^5^. Meanwhile, the use of 10% HP has been seen to produce effective whitening with no toxic effects on the pulp cells when applied for short periods of time. However, the application of gels containing 17.5% HP was toxic^9,25^.

The potential risks to the pulp tissue mean that more *in vitro* studies are necessary to assess the effects of whitening products used in clinical practice and to determine whether products containing identical or similar HP or CP concentrations lead to similar pulp cell behavior, regardless of the manufacturer.

For this reason, the present study analyses the diffusion capacity and possible cytotoxic effects of four commercially available whitening products commonly used in clinical practice. The null hypothesis tested in this study was that bleaching products containing an equivalent HP concentration but manufactured by different companies, would promote similar responses in DPSCs.

## Results

### Analysis of diffusion of hydrogen or carbamide peroxides

First, we analyze whether the different bleaching products display a different HP diffusion. Here, we demonstrated that there are some variations in the homovanillic acid dimer (HD) formation when compared the different bleaching products. As shown in Table 1, HD levels with Nor-CP were reduced compared to the observed with Opal-CP, Nor-HP and Opal-HP products. The Ultradent products exhibited higher diffusion than the Normon products.

**Table 1:** Mean diffusion values (95% confidential interval) in nmols/cm^2^.

	Diffusion
Nor-CP	66,4 (60,2–74,8)^a^
Opal-CP	70,9 (58,6–82,5)^b^
Nor-HP	70,2 (60,2–80,9)^b^
Opal-HP	85,8 (78,3–94,9)^c^

The same upper letter expresses groups without significant differences.

### Analysis of *h*DPSCs immunophenotype and multilineage differentiation potential

**Figure 1:**
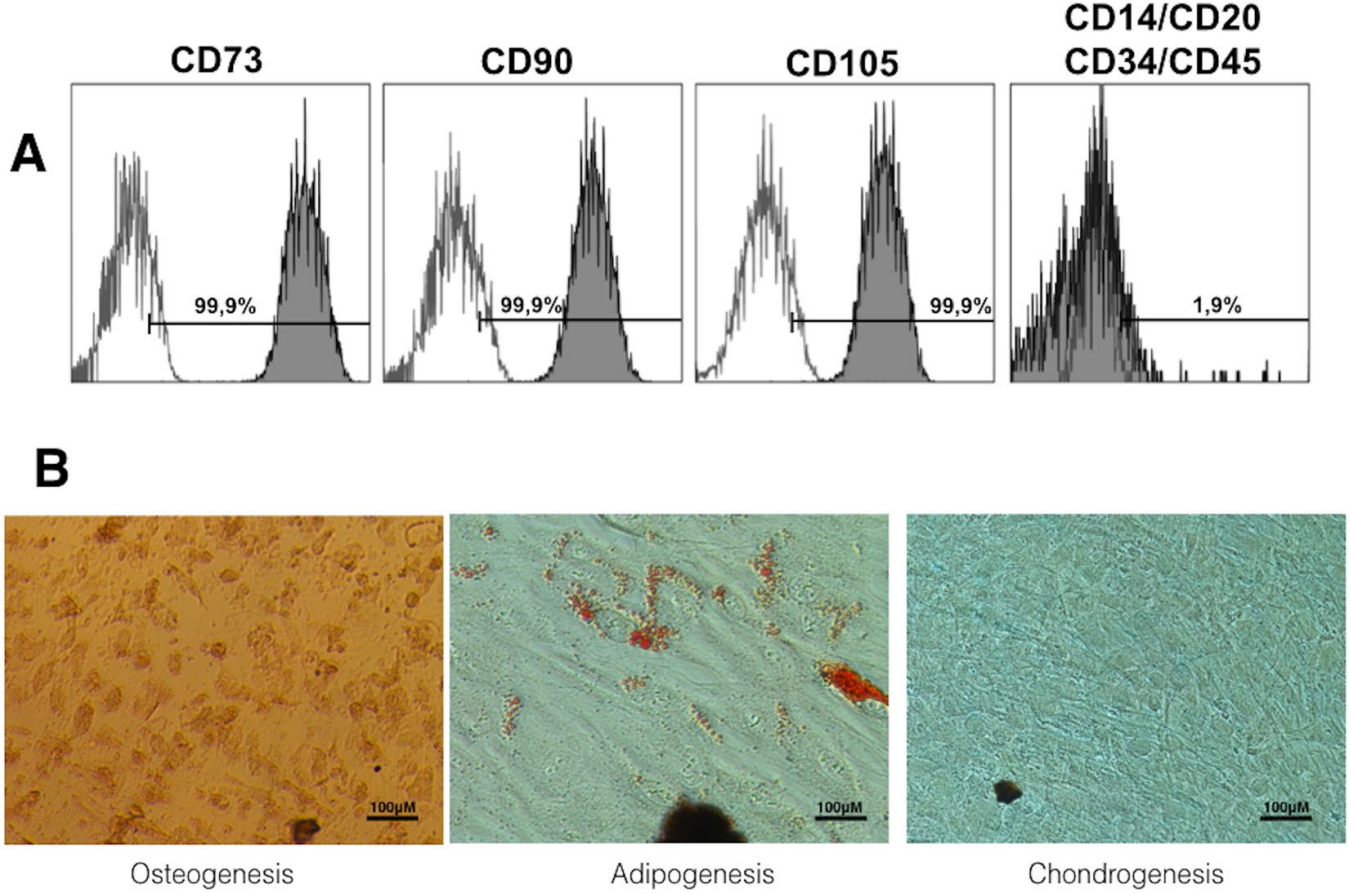
(**A**) Mesenchymal expression of Human DPSCs. Flow cytometry revealed high expression of human CD73, CD90 and CD105 and low expression of the haematopoietic markers CD14, CD20, CD34 and CD45. Numbers inside histogram represent the mean fluorescence intensity (MFI) values of only viable cells. Representative flow cytometry results are shown. (**B**) *In vitro* multipotential capacity of *h*DPSCs toward the osteogenic, adipogenic and chondrogenic lineages was analyzed.

Expression of the typical surface mesenchymal stem cell antigens CD73, CD90 and CD105, as well as the negligible expression of the haematopoietic CD34, CD45, CD14 and CD20 were analyzed on *h*DPSCs by flow cytometry. Most viable *h*DPSCs (>95%) displayed positive and negative expression of the mesenchymal and haematopoietic antigens, respectively (Fig. 1A). *h*DPSCs also showed multipotent differentiation potential to mesodermal lineages, such as adipocytes, chondroblasts and osteoblasts (Fig. 1B).

### MTT assays

**Figure 2:**
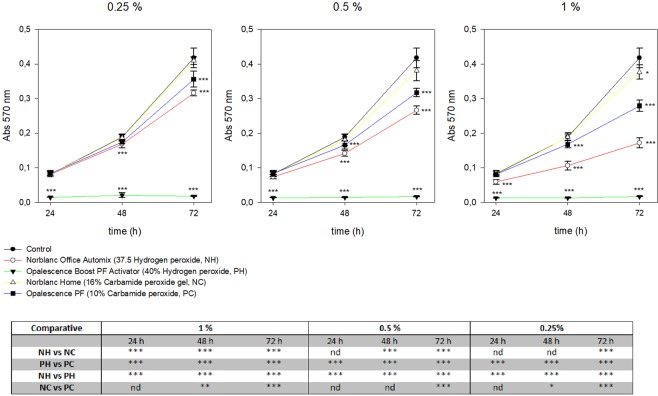
Cellular viability of *h*DPSCs exposed to bleaching products was measured by MTT assay. *h*DPSCs were treated with three different dilutions of Opal-HP, which yield a significant decrease in cell viability rates compared with the control group (***p < 0.001). In contrast, 0.5% and 0.25% dilutions of Nor-CP showed a slight recovery of cell viability. Some differences between peroxide bleaching products were obtained; Nor-H displayed a better cell viability rates than Opal-HP at 24, 48 and 72 h (***p < 0.001).

After, to evaluate the impact of the different bleaching agents eluates on the *h*DPSC viability MTT assays were performed (Fig. 2). Human Opal-HD-treated *h*DPSCs showed remarkable decreased cell viability in comparison to the control conditions (***p < 0.001). However, 0.5% and 0.25% of Nor-CP, Opal-CP and Nor-HP led to a slight recovery of cell viability compared with Opal-HP. Our results indicate that there are some differences between the analyzed peroxide bleaching products; Nor-HP displayed better cell viability than Opal-HP (***p < 0.001) at 24, 48 and 72 h. However, among the carbamide bleaching products, reductions in cell viability rates were significant at 72 h (***p < 0.001), but less evident after 48 h (*p < 0.05 or **p < 0.01) and not significant after 24 h.

### Effect of the treatment with bleaching extracts on cytotoxicity of *h*DPSCs

#### Measurement of reactive oxygen species

**Figure 3:**
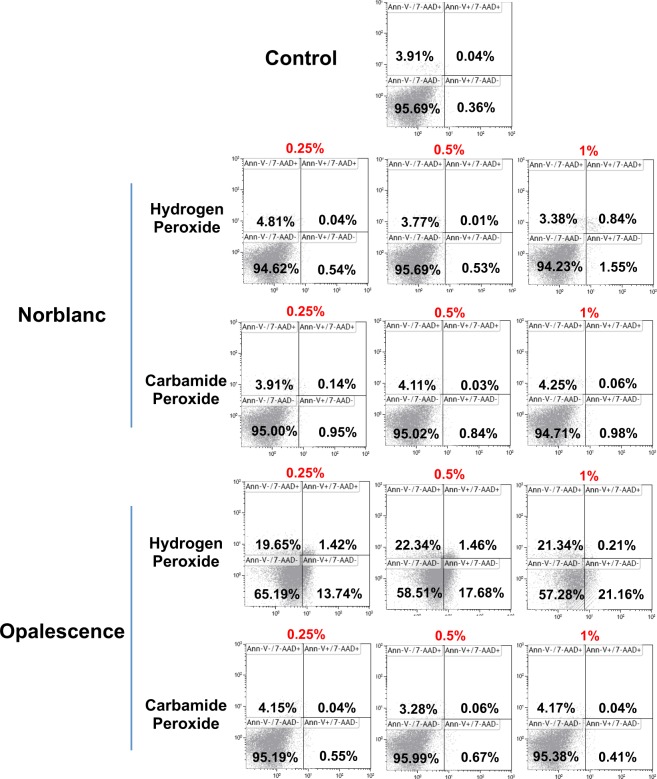
Human DPSCs were cultured in presence or absence (control) of the different bleaching extracts for 72 h, double-stained with Annexin-V and 7-AAD, and analyzed by flow cytometry. Numbers inside dot plots represent the percentages of live (bottom left quadrant), early apoptotic (bottom right quadrant) or late apoptotic and necrotic (upper left and right quadrants) cells. Representative flow cytometry results are shown.

**Figure 4:**
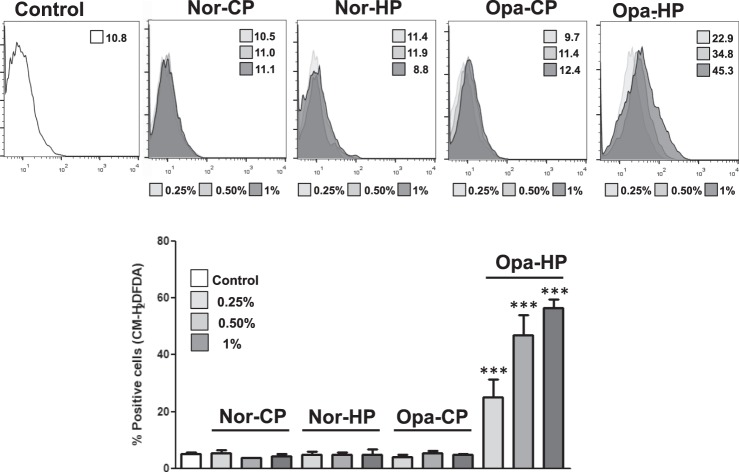
(**A**) Intracellular ROS levels was evaluated in untreated *h*DPSCs (control) or treated with several dilutions of Nor-CP, Nor-HP, Opal-CP, Opal-HP for 72 h by flow cytometry using the probe CM-H_2_DCFDA (5 μM). Numbers represent the mean intensity values obtained in each experimental condition. Representative histograms from three independent experiments are shown. (**B**) Percentages of positive cells for ROS production in each experimental condition are shown. Data are presented as mean ± standard deviation. Percentage of positive cells for ROS production was significantly increased compared to control cells, ***p < 0.001.

To determine whether the different bleaching products possess cytotoxic effects on *h*DPSCs, we studied the induction of cell death on *h*DPSCs after exposition with the distinct bleaching eluates by analyzing Annexin-V and 7-AAD staining, an analysis frequently employed to distinguish between necrotic and apoptotic cells. As we shown in Fig. 3, Opal-HP eluate-treatment for 72 h induced a reduction of viable cells (<66%), whereas in the presence of different dilutions of Opalescence CP, Norblanc HP and Norblanc CP live cells percentages were ≥93% and equivalent to those displayed by *h*PDLSCs cultured without any eluate (control). Similarly, a significant higher intracellular ROS level was measured in *h*DPSCs after exposure to Opalescence HP, mainly when using the more concentrated dilution, and compared to ROS levels detected in control cells or cells treated with Opalescence CP, Norblanc HP or Norblanc CP (***p < 0.001) (Fig. 4A,B).

### Immunocytofluorescence staining

**Figure 5:**
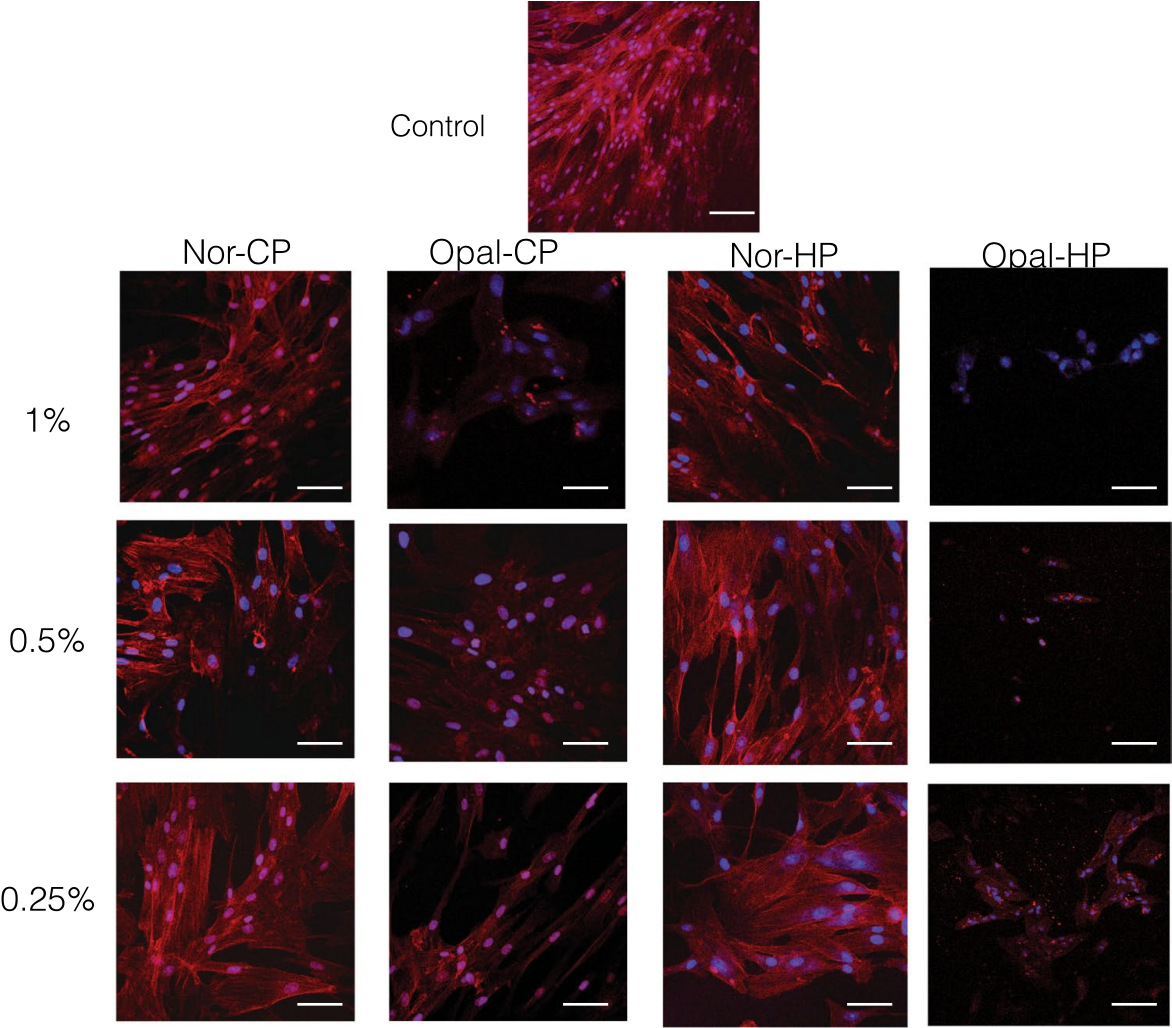
Immunofluorescence staining. hDPSCs were exposed to bleaching products extracts for 24 h. Then, F-actin fibers were visualized by CruzFluor594-conjugated phalloidin (red) with nuclei staining with 4,6-diamidino-2-phenylindole dihydrochloride (DAPI) (blue). Scale bar = 150 μm.

To address whether bleaching products affects cytoskeletal changes, we examined *h*DPSCs actin stress fiber formation using a confocal microscopy. Control *h*DPSCs displayed a gradual growth increase, an elongated and fibroblastic spindle-shaped morphology, and high content of F-actin (Fig. 5). Importantly, after treatment with 1% Opal-HP or Opal-CP, we found a certain percentage of cells showing no patent F-actin fibers and a condensed or fragmented nuclei, a common feature of apoptotic cells. However, Normon products, mainly the 0.25% solutions, displayed a similar organization and assembly of F-actin stress fibers in comparison to the control conditions^25^.

### *In vivo* analysis

**Figure 6:**
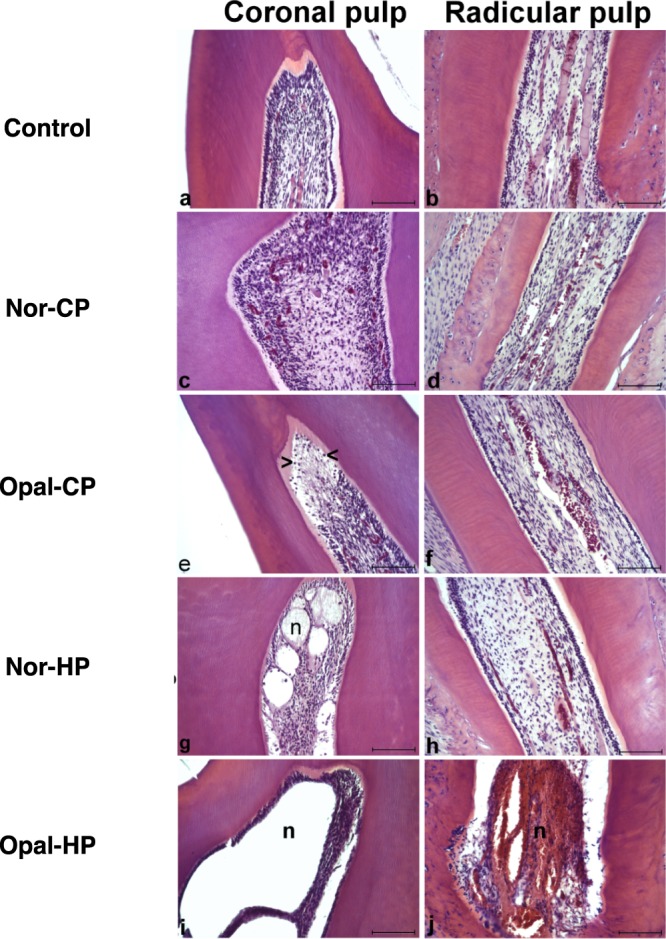
Representative images of coronal and radicular pulp from specimens of untreated control (**a**,**b**), Nor-CP (**c**,**d**), Opal-CP (**e**,**f**), Nor-HP (**g**,**h**) and Opal-CP (**i**,**j**) treated rats. While treatment with Nor-CP did not induced any histological lesion, treatment with Opal-CP induced the presence of mild inflammatory infiltrate in coronal pulp (**e**, head arrows). In the other hand, the addition of Nor-HP induced areas of necrosis on coronal pulp (**g**,**n**), and the treatment with Opal-HP induced a severe necrosis either in coronal (**i**,**n**) and radicular (**j**,**n**) pulp. Hematoxylin and eosin stain. Scale bar: 100 μm.

Histopathological examination revealed the lack of any significative pathological feature both control and Nor-CP specimens (grade 1, Fig. 6a–d). By the other hand, examination of specimens treated with Opal-CP revealed a mild inflammatory infiltrate (grade 2, mainly composed by macrophages and polimorphonuclear neutrophils) in coronal pulp of 2 specimens (Fig. 6e), while no lesions were observed in radicular pulp. The treatment with Nor-HP induced several areas of necrosis in coronal pulp in 2 specimens (grade 5, Fig. 6g), but newly, no lesions were evidenced in radicular pulp. These results contrasted with the specimens treated with Opal-HP, in which a severe necrosis both coronal and radicular pulp was observed in all specimens (grade 5, Fig. 6i,j).

## Discussion

A large number of reports have focused on the study of the biocompatibility of several bleaching agents, but with commercial products the investigations are more limited^3,15,24,26–28^. Moreover, the exact compositions of clinically available commercial bleaching products are often not completely specified, and they have provided different biocompatibility results in both *in vitro* and *in vivo* approaches^3,13,15,29^.

In this study the null hypothesis was rejected because the diffusion and biological effects in dental pulp stem cells were influenced by the brand in question.

The study evaluated four commercially available bleaching products, Nor-HP, Nor-CP, Opal-HP and Opal-CP, which were tested to evaluate their biological effects on *h*DPSCs. These cells were used because an inflammatory response, associated with local tissue necrosis, has previously been demonstrated after the application of high concentration HP bleaching gels (35–38%) to human teeth in *in vivo* models^6,30^. Moreover, this type of cell is easily obtained from human teeth, renews itself and has had the ability to differentiate into many cell types^31^. A further advantage is that the patient’s own cells can be used to test the action of new products^32,33^. Lastly, cells from different tissues frequently show different responses in toxicity tests^34^.

The high diffusion of bleaching products has been associated with high levels of cytotoxicity^35^, as, indeed, was found in our study. Another finding was that there was no correlation between the amount of hydrogen peroxide reaching the artificial pulp chamber and the concentration of the same in the product, so it is apparent that the diffusion as well as the toxicity is influenced by other products included in the final trade presentation. The obtained data point to lower diffusion and toxicity in Normon products than in Ultradent products. We found that Nor-CP displayed lower diffusion than Opal-CP despite containing the same concentration, which may be attributed by the effect of other non-bleaching components of the commercial product: stabilizers, thickeners, dyes, preservatives, even the viscosity of the gel that reaches the dental pulp. As all the bleaching products used in our study have a neutral pH, the effect of pH on the kinetics of decomposition and on the type of free radicals produced should not be considered a determinant factor in the diffusion process. Surprisingly, Nor-HP exhibited similar diffusion to Opal-CP, despite the different concentration of peroxide in both products.

A previous report demonstrated that the HP reaching the pulp chamber is more influenced by the bleaching protocol and the presence of other components, such as calcium, than by the concentration of HP^36^. The Ultradent-manufactured bleaching products used in our study contain potassium nitrate and fluoride and, to the best of our knowledge, the effect of these two components on HP diffusion has not been studied.

The MTT assay and the Annexin-V and 7-AAD staining were chosen to analyze the cell viability and apoptosis/necrosis, respectively. MTT is a well-known and accepted cytotoxicity assay^25,37^, in which viable cells reduce the MTT tetrazolium salt to a blue and insoluble product, formazan, which precipitates in the cytoplasm. The advantages The MTT assay and Annexin-V and 7-AAD staining were chosen to analyze cell viability and apoptosis/necrosis, respectively. MTT is a well-known and accepted cytotoxicity assay^25,37^, in which the MTT tetrazolium is reduced to formazan, which, in turn, precipitates in the cytoplasm. The advantages of this assay are its simplicity, accuracy, speed and the fact that there is no need to use a radioisotope^38^. In the presence of Opal-HP, MTT pointed to a significant reduction (***p < 0.001) in cell viability in comparison to the control conditions. This phenomenon was practically absent in the presence of Nor-CP, when cell viability rates were similar to those of the control group. In contrast, Opal-CP produces a greater reduction than Nor-CP in cell viability, despite having the same CP concentration. Previous studies reported no cell viability reduction when low concentrations of CP or HP were used^3,39^. However, Soares *et al*.^9^ reported a significant cell viability reduction in an enamel/dentin model, similar to that used in our study, in the bleached groups with a 17.5% HP gel, relative to control time-dependent.

Annexin-V is a protein member of the annexin family that binds to translocated inner plasma membrane phosphatidylserines, an early event that occurs during cell apoptosis^40^. Thus, Annexin-V together with a DNA binding compound such as 7-AAD is a common flow cytometry staining to distinguish between early or late apoptotic and necrotic cells. Our results revealed that Nor-CP, Nor-HP and Opal-CP did not induce cell death or apoptosis; however, Opal-HP was cytotoxic and provoked cell apoptosis and necrosis, a phenomenon that was concomitant with an increase in intracellular ROS levels. Similarly, Benetti *et al*.^35^ observed a concentration-dependent effect of HP on pulp tissue – a high concentration inducing necrosis and apoptosis, and a low concentration leading to a moderate inflammatory response, cell proliferation and apoptosis. However, our results here show that Nor-HP has a behaviour more similar to that of Opal-CP and Nor-CP than to the Opal-HP regardless of the peroxide concentration. Furthermore, Immunocytochemical assay evidenced that undiluted extracts of Normon products produced no cell morphological alterations or changes in their cytoskeletal organization pattern, while cells treated with other whitening products did produce such changes. As far as we know, this is the first study to make such an assertion possible.

Also, the biocompatibility of these bleaching products was investigated. Although our study used a non-human experimental model, previous reports found no differences between rats and human incisors, where a concentrated bleaching gel caused a substantial inflammatory reaction or pulp necrosis, corroborating the present results^18^. Indeed, Cintra *et al*.^15^ observed a major inflammatory response in the pulp tissue of rats after dental bleaching, just as we observed that Opal-HP produced necrosis on coronal and radicular pulp.

In summary, this study shows that similar concentrations of CP or HP in commercial whitening products may have different biological effects on hDPSCs and dental pulp tissue, suggesting that non-specified agents in bleaching products in their composition could influence their level of diffusion and/or cytotoxicity.

## Materials and Methods

### Sample preparation

Human third molars from 18–35 years-old healthy patients were extracted (n = 10). This procedure was approved by the Ethics Committee of our Institution (University of Valencia, registration number: H1443515306255). Additionally, all methodology used was carried out in agreement with the national clinical guidelines.

Teeth were washed to remove some organic remains, immersed in a 0.1% thymol solution at 4 °C for 48 h and finally placed in sterile distilled water at r/t until be used.

As we reported previously, pieces of 2-mm of teeth enamel and 2-mm of dentin (n = 20) were made and a mesiodistal cut was performed using a diamond disk. After, teeth roots were removed and the dentin surface was reduced with 400 and 600 grit silicon carbide paper Sof-lex™ discs (3 M Dental Products, St Paul, MN, USA) until the dentin thickness was 2-mm. Sample sizes ranged from 0.5 cm−0.7 cm × 0.4 cm–0.6 cm. After, the dentin surface was treated with 0.5 M EDTA in PBS pH 7.4 for 30 sec, to remove the smear layer without opening dentinal tubules diameter^25^ and stored in bidistilled water at 4 °C until be used. Before start experimental process, samples were randomly divided in four groups (n_i_ = 5).

As reported previously by our group, an artificial pulp chamber, with a capacity of 100 μL, was prepared with heavy silicone (Panasil R Putty, Kettenbach, Huntington Beach, CA, USA). A heavy silicone ring was fabricated to anchor the sample, allowing the dentin to be in contact with the buffer and had an upper window where to place the study gel. This ring fit perfectly in the artificial pulp chamber that maintained fixation of the sample; additional sealing was performed between the sample and the ring by using wax^25^.

### Evaluation of diffusion

Four commercial bleaching products manufactured by two different companies were evaluated. Normon products: neutral pH gel of 37.5% content of hydrogen peroxide (HP) Norblanc Office (Nor-HP) or with 16% carbamide peroxide (CP) Norblanc Home (Nor-CP) (Laboratorios Normon, S.A., Madrid, Spain). Ultradent products: neutral pH gel of 40% HP Opalescence Boost 40% PF (Opal-HP) or 16% CP Opalescence PF 16% (Opal-CP) (Ultradent Products, Inc, South Jordan, UT, USA).

Analysis diffusion of HP from the studied bleaching compounds was carried out by fluorimetric techniques. Measurement of homovanilic acid dimer formation from a reaction catalysed by peroxidase using HP as a substrate was measured using a fluorimeter (model F-4500 fluorescence spectrophotometer, Hitachi, Japan). Previously, we made a standard fluorimetry signal curve for HP (H1009-100ML, Sigma-Aldrich). Alternatively, same dilutions were analyzed in a spectronic Helios alpha double-beam UV-Visible scanning spectrophotometer at 240 nm (Thermo Fisher Scientific Inc., Waltman, MA, USA). After, the fluorimetry data was related to the fluorescent dimer concentration.

After, a volume of 400 μL of PBS was added in the reservoir and 5 μL of the bleaching gel was applied to the external teeth enamel surface. The HP was maintained for 30 min in the Nor-HP and Opal-HP groups and for 90 min in the case of Nor-CP or Opal-CP gels. All the bleaching process was carried out at 37 °C.

Finally, a volume of 100 μL of diffused HP-containing sample was taken out and diluted with 1400 μL of the reaction buffer and diluted again with and additional volume of 500 μL glycine-EDTA buffer after 15 min. Finally, concentration values were calculated after extrapolating fluorescence intensity data into the standard curve, as reported^25^.

### Biological assays

#### *h*DPSCs isolation and characterization

Human DPSCs (*h*DPSCs) were isolated from extracted third molars from healthy donors (n = 10) as reported^41,42^. Written informed consent was obtained from donors in accordance with Helsinki declaration guidelines and Ethics Committee of our Institution (University of Murcia; ID: 1417/2016). First, dental pulp was extracted from the pulp chamber and root canals, extensively rinsed with Hank’s balance salt solution and submitted to enzymatic digestion with 3 mg/mL collagenase A (Roche Diagnostics, Basel, Switzerland) for 1 h at 37 °C. After, the obtained cells were seeded into 75-cm^2^ culture flasks (Corning, New York, USA) and cultured in Alpha-MEM medium containing 100 U/mL penicillin and streptomycin and 10% fetal bovine serum (FBS) in an incubator at 37 °C and 5% CO_2_. Human DPSCs used for the subsequent experiments were from passage 4 onwards.

After, cultured *h*DPSCs was analyzed by flow cytometry using specific anti-human antibodies against CD73, CD90, CD105, CD34, CD45, CD14 and CD20 (Human MSC Phenotyping Cocktail, Miltenyi Biotec, Bergisch Gladbach, Germany), following the criteria recommended by the International Society for Cellular Therapy (ISCT)^43^. Also, the mesodermal differentiation potential of *h*DPSCs toward the adipogenic, osteogenic and chondrogenic lineages was analyzed. Adipogenic differentiation was conducted after culturing cells in StemMACS AdipoDiff media (Miltenyi Biotec) for 14 days. Then, *h*PDSCs were fixed with 4% paraformaldehide (PFA) and stained with Oil Red O solution (Sigma-Aldrich, St. Louis, MO, USA) to analyze formation of neutral lipid-containing cytoplasmic vacuoles. To assess osteogenic differentiation potential, *h*PDSCs were cultured in StemMACS OsteoDiff media (Miltenyi Biotec) for 21 days. Then, cells were fixed with 4% PFA and stained with Alizarin Red solution (Sigma-Aldrich, St. Louis, MO, USA) to analyze the formation of calcium-rich deposits in the cultures. Finally, chondrogenic differentiation was induced using StemMACS CondroDiff media (Miltenyi Biotec) for 21 days. Cells were fixed as above and analyzed with Alcian blue staining (Sigma-Aldrich) to detect mucopolysaccharides and acidic mucins.

### Conditioned medium

For obtention of conditioned culture medium (CM) containing the bleaching products, we followed a protocol previously described^44^ and in compliance with the ISO 10993-12 procedure. In brief, 100 μL of the diffused product sample was taken out and diluted to 1%, 0.5% and 0.25% with culture medium. After, CMs were filtered and stored until be used in the different assays.

### Metabolic activity assay

The cytotoxicity of the extracts to the stem cells was assessed using the MTT assay (MTT Cell Growth Kit, Chemicon, Rosemont, IL, USA), as reported previously^25^. Briefly, *h*DPSCs were seeded at 1 × 10^4^ cells/well in a volume of 180 μL DMEM medium w/o phenol red in 96-well plates. Previously, *h*DPSCs were starved for 24 h in serum-free culture medium at 37 °C and subsequently exposed the different bleaching eluates. Cells cultured in Alpha-MEM medium plus 10% FBS served as positive control. A final concentration of 1 mg/mL MTT was added after 24, 48 and 72 h of culture and incubated for 4 h. Finally, the MTT-containing medium was removed, and 100 µL of dimethyl sulfoxide (DMSO) was added to solubilize formazan. Absorbance at 570 nm (Abs_570_) was determined, using Abs_690_ as the reference wavelength. Each condition was performed in triplicate.

### Analysis of cell viability by flow cytometry

To analyze cell viability of *h*DPSCs after treatment with the different bleaching extracts for 72 h, cells were stained with Annexin-V and 7-AAD (Immunostep, Salamanca, Spain) following manufacturer’s instructions. Subsequently, percentages of live, early or late apoptotic and necrotic cells were determined in a flow cytometer.

### Measurement of intracellular ROS

The production of ROS (reactive oxygen species) was analyzed using the cell-permeable general oxidative sensor 5-(and-6)-chloromethyl-2′,7′-dichlorodihydrofluorescein diacetate (CM-H_2_DCFDA) (Molecular Probes, Eugene, OR, USA). Briefly, *h*DPSCs were incubated with 5 μM CM-H_2_DCFDA at 37 °C for 30 min. After, intracellular ROS generation was evaluated by flow cytometry in living cells.

### Immunocytochemical staining

Phalloidin staining and nuclear labeling with DAPI were performed as previously described^25^. For determine the effects of bleaching products on cell morphology, *h*DPSCs were adhered on glass coverslips in culture medium containing the different material extracts. Briefly, cells were fixed in 4% paraformaldehyde in PBS for 20 min at 37 °C, permeabilized with 0.25% Triton X-100 (Sigma-Aldrich) in PBS for 3 min, and stained with CruzFluor594-conjugated phalloidin (Santa Cruz Biotechnology, Dallas, TX, USA). Also, nuclei were labelled with 4,6-diamidino-2-phenylindole dihydrochloride (DAPI) (Sigma-Aldrich). Finally, fluorescence micrographs were acquired using a fluorescence confocal microscopy (Zeiss, Oberkochen, Germany).

### Animals

Rats were supplied by the Animal Care Facility from the University of Murcia (Spain) (REGA ES300305440012). All procedures involving the use of animals were previously approved by the Bioethics Committee from the University of Murcia and the pertinent competent authority (A1320141001), and followed European Union guidelines for animal experimentation (EU/63/2010).

A total of 15 male Wistar rats with a mean weight of 350 g, were employed in the *in vivo* study. Rats were kept in a standard animal room at 22 ± 1 °C, 55 ± 10% humid atmosphere, 12-hour light-dark cycle, and free access to food and water *ad libitum*.

Five groups of n = 3 animals per group were used: i) Opal-HP, ii) Nor-HP, iii) Opal-CP, iv) Nor-CP and v) placebo gel. The bleaching gel (0.01 mL) was applied to the surface of the molars^13^ according to the manufacturer’s recommendations. The animals were anesthetized by intraperitoneal administration of ketamine (Ketavet 100, Gellini Farmaceutici Spa, Peschira Borromea-MI, 32 mg/kg) and xylazine (Rompun, Bayer AG, Leverkusen-Germania, 20 mg/kg).

Two days after the application of the dental bleaching treatment, animals were sacrificed, the hemi-maxillae was fixed in 4% neutral buffered formalin (Panreac Quimica, Barcelona, Spain) for 24 hours, and immersed for one week in a 10% aqueous formic acid commercial solution (TBD-2, Thermo Corp., Madrid, Spain) for bone decalcification. After, bone samples were embedded in paraffin. Three micrometers sections were then obtained for all specimens, and stained with a standard hematoxylin and eosin staining for routine histopathological examination.

To determine the degree of damage induced by the bleaching procedures in dental pulp, a previously proposed scale was used^15^. This semi-quantitative scale establish a score according to the relative numbers of inflammatory leukocytes per high power field (HPF) (400x), and comprised 5 degrees: grade 1 (absence of inflammatory infiltrate or negligible in number), grade 2 (mild inflammatory infiltrate, *i*.*e*. <25 cells/HPF), grade 3 (moderate inflammatory infiltrate, *i*.*e*. 25–125 cells/HPF), grade 4 (severe inflammatory infiltrate, *i*.*e*. >125 cells/HPF) and grade 5 (necrosis). All examinations were performed by using an upright light microscope (Zeiss Axio Scope A10, Zeiss, Madrid, Spain), with a digital camera (Axio Cam Icc3, Carl Zeiss, Jenna, Germany), by using a specific digital analysis software (AxioVision ver. 4.9.1, Zeiss, Jenna, Germany).

### Statistical analysis

MTT assay, apoptosis and diffusion capacity results were analyzed using SPSS version 22.0 statistical software (SPSS, Inc., Chicago, IL, USA). All experiments were conducted in triplicate and repeated at least twice. Data of absorbances are shown as the mean ± standard deviation (SD). Comparisons between groups were analyzed using one-way ANOVA test followed by a Bonferroni post-test for multiple comparisons A *p* value < 0.05 was deemed significant (*p < 0.05, **p < 0.01, ***p < 0.001).

## Conclusion

In general, our results showed that Ultradent products exhibited a higher diffusion than Normon products. Of note was the fact that Nor-CP resulted less cytotoxic than the other commercial bleaching products assayed.
